# Effectiveness and cost savings of employing speech therapists in a home artificial nutrition unit

**DOI:** 10.3389/fresc.2025.1553818

**Published:** 2025-08-20

**Authors:** Alice Di Battista, Ylenia Di Giancaterino, Lucia Di Palma, Claudia Nunziata, Loredana Gigli, Antonio Vivenzio, Alessandra Matano

**Affiliations:** ^1^ Centro Paraplegici Ostia, UOC Riabilitazione e Centro Spinale, Asl Roma 3, Rome, Italy; ^2^ Ordine TSRM PSTRP Roma e Provincia, CdA Logopedisti, Rome, Italy; ^3^ Rehabilitation Health Care Professionals, Asl Roma 3, Rome, Italy; ^4^ Homecare Assistance and Home Artificial Nutrition Unit, Asl Roma 3, Rome, Italy; ^5^ Dipartimento Salute Mentale, UOC TSMREE, Asl Roma 3, Rome, Italy

**Keywords:** speech language pathologist (SLP), artificial nutrition (AN), home artificial nutrition, thickener, water gel

## Abstract

The Home Artificial Nutrition Unit (HANU) deals with both dysphagic patients receiving enteral and parenteral nutrition and patients who can eat orally with restrictions. In the Lazio Region, the HANU prescribes water gels and thickeners, supplied by the National Health Service (NHS), for safe hydration. Before the employment of a Speech and Language Pathologist (SLP) in the HANU (January 2023), prescriptions were standardized, regardless of the swallowing impairment severity: four jars of thickeners per patient/month and six water gels daily. The Clinical Swallowing Evaluation (CSE) made by the SLP allowed to customize the amount of thickeners and water gels based on the actual needs of the patient. The aim of this study is to investigate the cost savings resulting from the employment of an SLP in the HANU. A descriptive retrospective study was conducted, which analyzed the SLP activity in the HANU of ASL Roma 3 (January–December 2023). The study group consisted of 149 patients (61 males, 88 females with a mean age of 76.2 years) with different pathologies. The cost of each product (thickeners and gelified water) was provided by the Regional Pharmacy. The annual savings were calculated as the difference between the standardized prescription of products (as usual care in our department) and personalized prescription after SLP assessment. Since January 2023, there has been an increasing trend in requests for SLP assessments. The employment of an SLP in the HANU resulted in an annual economic saving of €30,278.67. This preliminary study shows how the employment of an SLP in the HANU can reduce inappropriate prescriptions of thickeners and water gels, thus ensuring cost savings.

## Introduction

The Home Artificial Nutrition Unit (HANU) deals with both dysphagic patients receiving enteral and parenteral nutrition and patients who can eat orally but with restrictions. These methods can improve clinical and quality of life (QoL) outcomes, but determining the most effective and safe methods can involve complex decision-making: healthcare professionals should consider using oral, enteral, or parenteral nutrition support, alone or in combination, for people who are either at risk of malnutrition or dehydration or for subjects who are unable to fully satisfy their nutritional needs (1). Artificial nutrition (AN) is often started during hospitalization and continues as long-term home therapy. Typically, there are only minor differences in the indication for Home AN (HAN) and for in-hospital AN. In HAN, additional criteria need to be considered carefully, such as prognosis, health-related QoL, and any ethical aspect of a treatment. To start HAN, the guiding principle is that without AN, the nutritional status of the patient is expected to deteriorate significantly, impacting their prognosis and QoL (2).

With regard to the Italian national prevalence, the most recent national survey carried out by the Italian Society for Artificial Nutrition and Metabolism shows that the number of recorded cases of HAN was 14,441 (90.3% adult and 9.7% pediatric patients) and the prevalence was 325.5/per million inhabitants. Among the disease categories in adult patients, oncological diseases accounted for 19.4% of the total HAN, neurological diseases 64.8%, gastrointestinal diseases (GI) 6.5%, and other diseases 9.3%. In pediatric patients, the main disease category distribution was GI disease (49.7%) and neurological pathologies (63.4%) (3).

Impaired swallowing can cause increased anxiety and fear: many patients avoid oral intake, leading to malnutrition, isolation, and depression, worsening their QoL. Understanding and balancing the risks and the potential benefits of continuing oral intake without restrictions or choosing to activate the HANU makes this a challenging area of healthcare (4).

In this regard, the importance of speech therapy–related activities such as assessment, intervention for safe swallowing, follow-up, and possible weaning practices of dysphagic patients should be highlighted (5). However, it should be underlined that regarding the presence and intervention of a Speech and Language Pathologist (SLP) in the HANU, scientific literature still struggles to show evidence on speech therapy intervention techniques and their effectiveness on the level of swallowing in the HAN service.

In a home setting, together with a multidisciplinary team, the SLP provides personalized support to the caregiver in acquiring skills, competence, and familiarity with the new indications for AN, especially to facilitate a continuation of oral intake, as appropriate. In fact, many patients need modified diet textures; for example, liquids may need to be thickened and/or foods may need to be pureed to prevent the patient from choking or contracting aspiration pneumonia (6, 7).

The healthcare costs related to dysphagia and its complications are substantial. Several studies have shown that individuals with dysphagia experience longer hospital stays, higher hospital readmission rates, increased mortality within a year of admission, and overall greater utilization of healthcare services (8, 9).

In the Lazio Region, the HANU prescribes water gels and thickeners, supplied by the National Health Service (NHS) (10), for safe hydration. The HANU of Local Health Unit “Roma 3” that covers an area of 605,534 inhabitants handles 1,570 patients. Before the employment of an SLP in the HANU (January 2023), prescriptions were standardized, regardless of the swallowing impairment severity: four jars of thickeners per patient/month and six water gels daily. The Clinical Swallowing Evaluation (CSE) made by the SLP allowed to customize the number of thickeners and water gels based on the actual needs of the patient.

The aim of this study is to investigate the cost savings resulting from the employment of an SLP in the HANU.

## Materials and methods

A descriptive retrospective study was conducted analyzing SLP activity in the HANU of Local Health Unit “Roma 3” from January to December 2023.

At first, we analyzed the different characteristics of patients for whom CSE was performed, such as age, sex, and pathologies; then the trend was determined and the total number of requests of SLP assessments in the HANU in 2023 was calculated.

There were no exclusion criteria for the selection of patients, since all patients assessed by a speech therapist through the HANU were included in the study. Therefore, the sample was also completely representative of each patient referred to this service, and it was not a convenience sample. The comparison method was therefore purely descriptive.

The cost of each product (thickeners and water gels) was provided by the Territorial Pharmacy, and from this cost was calculated the annual savings accruing from personalized prescription after SLP assessment.

## Results

The study group consisted of 149 patients: 61 males – 41%; 88 females – 59%, with a mean age of 76.2 years (minimum age 31, maximum age 100) with different pathologies.

**Figure 1:**
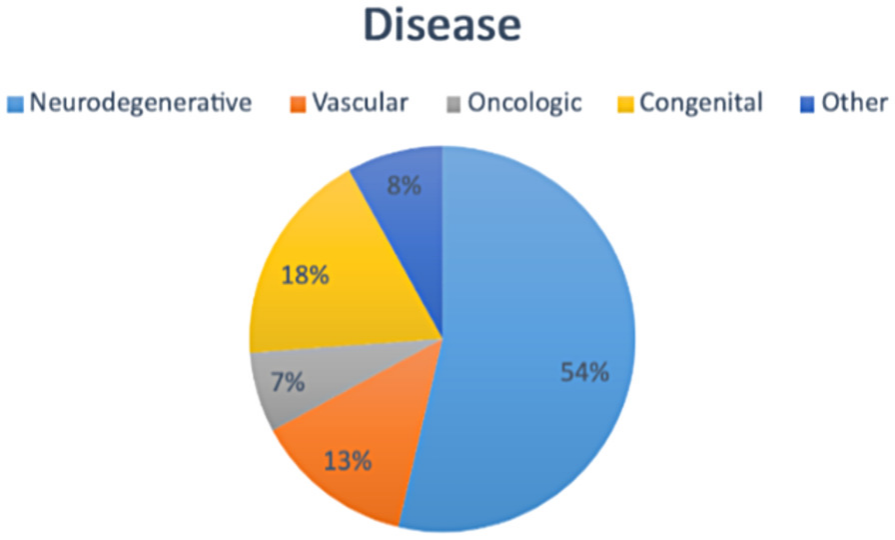
Frequencies of disease in SLP assessment in the HANU (149 patients).

In Figure 1, we report the most common pathologies found in patients evaluated by the SLP. There were neurodegenerative diseases in 54% of them (amyotrophic lateral sclerosis, multiple sclerosis, Alzheimer's, Parkinson's disease and parkinsonism, cognitive impairment), followed by 18% of congenital diseases (PCI, Down Syndrome, and Rett Syndrome); 13% of vascular diseases (ischemic and hemorrhagic stroke); 7% of oncological diseases (head/neck tumors); and 8% of other etiologies (cranioencephalic trauma, respiratory infections, and cardiac pathologies).

**Figure 2:**
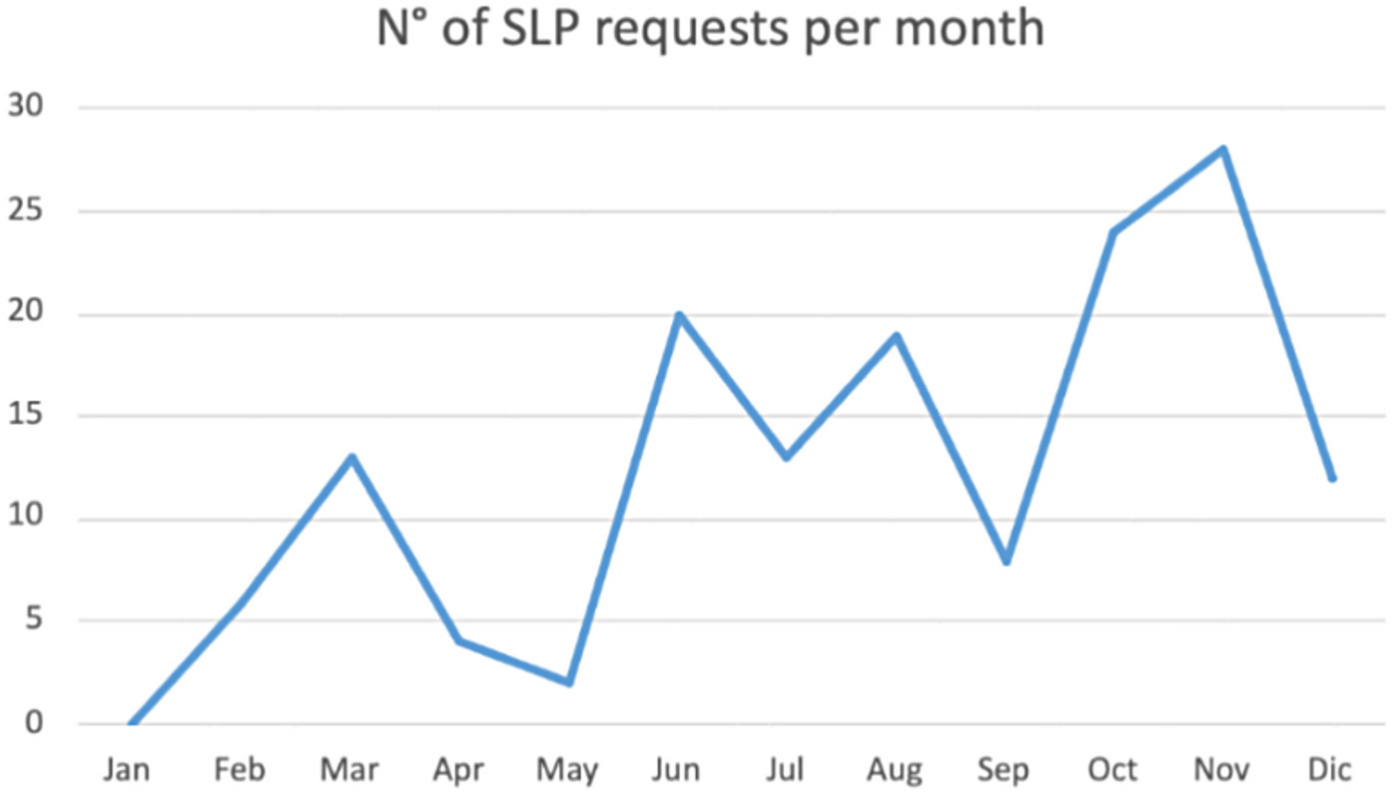
Trend in requests for SLP assessments in 2023.

Since January 2023, there has been an increasing trend in requests for SLP assessments with a peak in November 2023, as can be seen in Figure 2.

**Figure 3:**
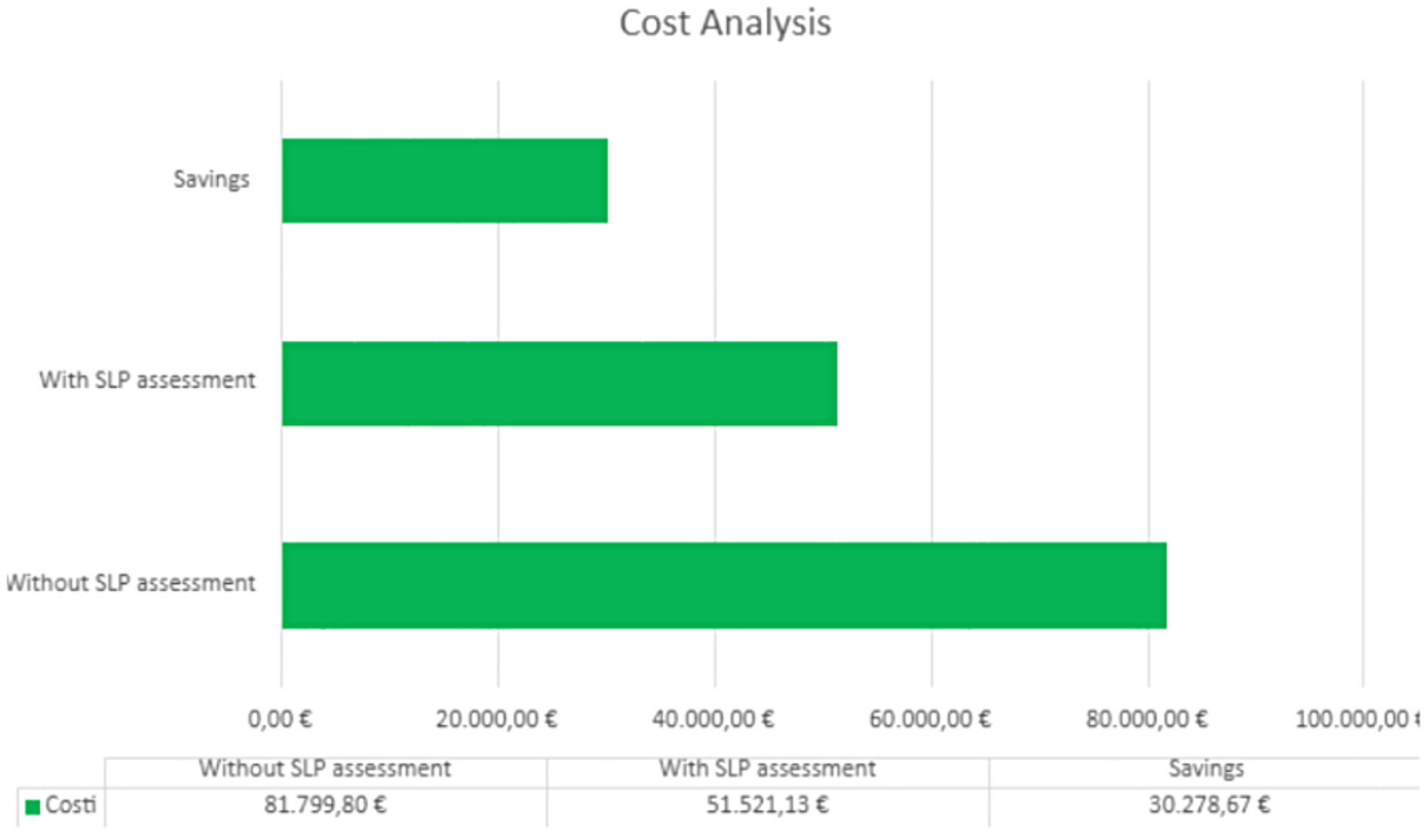
Annual costs analysis with and without SLP assessment of thickeners and water gels.

The annual savings were calculated as the difference between the standardized prescription of products (as usual care in our department) and personalized prescription after SLP assessment in the 149 patients who were evaluated. Considering that the cost of the standardized prescription for each patient, before the introduction of the speech therapist in the HANU, was approximately €1221 per month for thickeners and €183 per day for water gels (the cost of each product was provided by the Territorial Pharmacy), from a comparison of these expenses with those actually incurred after speech therapy assessments and related personalized prescriptions, an annual saving of €30,278.67 was recorded (Figure 3).

## Discussion

This preliminary study shows how the employment of an SLP in the HANU can reduce inappropriate prescriptions of thickeners and water gels, thus ensuring cost savings.

Before the employment of an SLP in the HANU team, the dosage of thickeners and water gels was standardized for every patient (four jars of thickeners per month, six water gels daily). After the employment of the SLP, the prescription began to be personalized according to the real needs of each patient. The SLP can suggest 0–6 jars of thickeners per month and 0–8 water gels daily. This variability in dosage is due to differences among patients; for instance, some have mixed hydration needs, requiring smaller amounts of these products. In addition, the required quantity of thickening agents varies depending on the severity of dysphagia. In this way, a controlled prescription of thickeners and water gels results in waste reduction and significant economic savings.

Furthermore, since the beginning of this project, there has been a positive trend in requests for SLP assessments. This phenomenon was correlated both to a structured and a constant SLP intervention in a home setting and to an active collaboration with General Practitioners who have shown to be sensitive to the problem of swallowing and responsive to this new service offered.

Finally, there is a cost-effectiveness saving arising from the role of the SLP in preventing dysphagia complications and hospitalization. Patients with mobility impairments receive SLP assessments directly at home. The possibility of receiving treatments directly at home indicates a significant improvement in QoL for both the patient and the caregiver. During dysphagia evaluation, the SLP gives instructions to the patient and the caregiver about correct feeding and hydration methods; this allows the prevention of dysphagia complications; also, the risk of hospitalization for aspiration pneumonia is reduced.

This study has some limitations that need to be considered when interpreting the results. First, the sample size is relatively small (149 patients), although it is representative of the users actually followed by the HANU during the observation period. A larger number of patients could have ensured greater statistical power and generalizability of the results.

A second limitation is the lack of a control group. The study includes only patients with dysphagia who were prescribed thickeners and water gels, making it methodologically irrelevant to establish a comparison group with nondysphagic subjects, to whom these products are not prescribed. As a result, it was not possible to directly compare costs with an untreated population. This limits the possibility of exclusively attributing the observed cost reduction to the intervention of speech therapists, although it suggests a significant role.

Finally, potential confounding factors, such as variations within different local health units in the Lazio region in prescribing patterns, product availability, or clinical practices, must be considered.

Although these aspects may influence economic analysis, detailed public data on regional average costs for thickeners and water gels are not available, which prevents a more in-depth comparative analysis and adjustment for these variables.

Despite these limitations, the results obtained offer useful insights into the possible impact of speech intervention in a HANU setting and open the way for larger, controlled future studies.

## Data Availability

The datasets presented in this article are not readily available because of restrictions. Requests to access the datasets should be directed to Alice Di Battista, alice.dibattista@aslroma3.it.

## References

[1] Shen Z Hou Y Huerman A Ma A. Patients with dysphagia: how to supply nutrition through non-tube feeding. Front Nutr. (2022) 9:1060630. 10.3389/fnut.2022.1060630 36532550 PMC9757495

[2] National Collaborating Centre for Acute Care (UK). Nutrition Support for Adults: Oral Nutrition Support, Enteral Tube Feeding and Parenteral Nutrition. London: National Collaborating Centre for Acute Care (UK) (2006). 21309138

[3] Pironi L, Regional Coordinators of SINPE. Development of home artificial nutrition in Italy over a seven-year period: 2005–2012. BMC Nutr. (2017) 3:6. 10.1186/s40795-016-0118-y

[4] Lisiecka D Kearns Á Bonass A. A qualitative systematic review of family caregivers’ experiences of artificial nutrition and hydration at home: a meta-ethnography. Int J Lang Commun Disord. (2022) 57(4):717–36. 10.1111/1460-6984.12726 35439344 PMC9543238

[5] Bai AV Agostini F Bernetti A Mangone M Fidenzi G D'Urzo R State of the evidence about rehabilitation interventions in patients with dysphagia. Eur J Phys Rehabil Med. (2021) 57(6):900–11. 10.23736/S1973-9087.21.06716-2 33541045

[6] Giles M Barker M Hayes A. The role of the speech-language pathologist in home care. Home Healthc Nurse. (2014) 32(6):349–53. 10.1097/NHH.0000000000000079 24887271

[7] Duncan S Menclova A Huckabee ML Cadilhac DA Ranta A. How much does dysphagia cost? Understanding the additional costs of dysphagia for New Zealand in patients hospitalised with stroke. Neuroepidemiology. (2024) 59(1):1–11. 10.1159/000539133 38718760

[8] Patel DA Krishnaswami S Steger E Conover E Vaezi MF Ciucci MR Economic and survival burden of dysphagia among inpatients in the United States. Dis Esophagus. (2018) 31(1):1–7. 10.1093/dote/dox131 29155982 PMC6454833

[9] Attrill S White S Murray J Hammond S Doeltgen S. Impact of oropharyngeal dysphagia on healthcare cost and length of stay in hospital: a systematic review. BMC Health Serv Res. (2018) 18(1):594. 10.1186/s12913-018-3376-3 30068326 PMC6090960

[10] Home Artificial Nutrition Path in the Lazio region. Decree 404 of 03/09/2013.

